# Editorial: Extracellular vesicles and miRNAs: pioneers in nutritional science and functional food development

**DOI:** 10.3389/fnut.2025.1624517

**Published:** 2025-06-16

**Authors:** Erika Cione, Diego A. Bonilla, Maria Cristina Caroleo, Diana Marisol Abrego-Guandique, Roberto Cannataro

**Affiliations:** ^1^Department of Pharmacy, Health and Nutritional Sciences, University of Calabria, Rende, Italy; ^2^Galascreen Laboratories, University of Calabria, Rende, Italy; ^3^Hologenomiks Research Group, Department of Genetics, Physical Antropology and Animal Physiology, University of the Basque Country (UPV/EHU), Leioa, Spain; ^4^DBSS Research Division, Dynamical Business Science Society–DBSS International SAS, Bogotá, Colombia; ^5^Grupo de investigacion NUTRAL, Facultad Ciencias de la Nutrition y los Alimentos, Universidad CES, Medelin, Colombia; ^6^Department of Health Sciences, University of Magna Graecia Catanzaro, Catanzaro, Italy

**Keywords:** nutrition, microRNAs, extracellular vesicles, milk, honey

MicroRNAs (miRNAs) are small nucleotide sequences (20–25 bases) capable of epigenetic action, in particular downregulation of protein synthesis; they have been seen for a long time as subproducts without a defined biochemical and physiological function. They are found in almost all biological fluids, in particular in the blood, and they circulate mainly through extracellular vesicles (EVs), of which the smaller dimensions are called exosomes (1).

Recent research has shown how they can be seen as markers of some pathological states. This would be of great interest, especially for pathologies with an uncertain diagnosis, such as lipedema, or for those who have an early diagnosis and act promptly (2).

Several miRNAs are highly conserved among species; hence, the hypothesis that foods of animal origin can be carriers of miRNAs, which could represent a direct epigenetic regulator. This hypothesis is fascinating for foods of animal origin. Even from humans, milk is particularly rich in EVs containing miRNAs with immunomodulatory properties for mammalian offspring. Notably, this may also be true for human adults, given that milk (from various mammals) and dairy are part of the diet of various populations worldwide (3–5).

In their review, Di et al. report a comprehensive picture of EV production and secretion, pointing out that they represent a proper micro-environment with different content of miRNAs but also DNA, proteins, and enzymatic complexes. EVs possess different receptors, such as CD36, CD9, and CD81, and integrins, so they can interact with other cells, bringing biochemical messages. The authors also analyze the different methods of isolating EVs, concluding that, at the moment, there is no gold standard. Finally, they highlight the decisive action of EV-derived miR on the immune system and the regulation of inflammation, focusing on NFkB and p53 pathways.

In the same direction, Xu et al. report different content of miRNA in mammals, emphasizing how milk from the giant panda contains more than 100 miRNAs, which is almost five times that of cow milk and double that of human milk, which has a very high content of immunoregulatory miRNAs. The difference in giant panda milk microRNA content has not yet been explained. However, an important feature of this work lies in the analysis of the bioavailability of miR, whose mechanism is not yet well understood, as well as it would be necessary to analyze better the treatments affecting foods, for example, the pasteurization of milk, to understand if this has a depressing action on bioavailability. However, from the literature, EVs-miRNA can be absorbed and exert a biological function.

Hsu et al., even in cell culture, show the action of EVs derived from kale juice, showing how this could affect downregulation of the Smad7 pathway, resulting in collagen production. Is this the first step to better understanding and cross-kingdom communication? EVs from fruits/plants could have an additive action on plant-derived substances with epigenetic action, such as polyphenols. A very promising food, especially regarding immune response and inflammatory status, is honey (6, 7). Used by humans for thousands of years, it has recognized beneficial properties, but has a mechanism that has never been fully elucidated; miRNAs could provide an answer in this sense.

Our group shows how even a diet, specifically ketogenic diets (a nutritional program that allows a maximum carbohydrate intake of 30 g per day), can affect miRNAs and how they act to regulate cognitive function. However, miRNAs could be a handy tool to evaluate the positive action of the diet itself, for example, in inflammatory status or in managing pathological conditions such as sarcopenia or lipedema (8, 9).

We can conclude that the impact of the EVs' microRNA cargo, contained in foods, on human health is still inconclusive (10) because of the limitations of the molecular biology techniques used to monitor them and/or the assessment of EVs' stability in the gastrointestinal tract. Future directions should focus on EV stability using INFOGEST 2.0 and the content consisting of conserved microRNA cargo, the creation of an experimental model using organs-on-chip methodology to mimic the gut environment, and the examination of the impact of the microbiome milieu on digesta EVs, elucidating whether or not microbes take EV-microRNA cargo, as is true for several polyphenols.

## References

[1] Doyle LM Wang MZ. Overview of extracellular vesicles, their origin, composition, purpose, and methods for exosome isolation and analysis. Cells. (2019) 8:727. 10.3390/cells8070727 31311206 PMC6678302

[2] Cione E Abrego Guandique DM Caroleo MC Luciani F Colosimo M Cannataro R. Liver damage and microRNAs: an update. Curr Issues Mol Biol. (2022) 45:78–91. 10.3390/cimb45010006 36661492 PMC9857663

[3] Ahlberg E Al-Kaabawi A Thune R Simpson MR Pedersen SA Cione E. Breast milk microRNAs: potential players in oral tolerance development. Front Immunol. (2023) 14:1154211. 10.3389/fimmu.2023.1154211 36999032 PMC10045994

[4] Sanwlani R Fonseka P Chitti SV Mathivanan S. Milk-Derived extracellular vesicles in inter-organism, cross-species communication and drug delivery. Proteomes. (2020) 8:11. 10.3390/proteomes8020011 32414045 PMC7356197

[5] Pietrzak-Fiećko R Kamelska-Sadowska AM. The comparison of nutritional value of human milk with other mammals' milk. Nutrients. (2020) 12:1404. 10.3390/nu12051404 32422857 PMC7284997

[6] Gismondi A Di Marco G Canini A. Detection of plant microRNAs in honey. PLoS ONE. (2017) 12:e0172981. 10.1371/journal.pone.0172981 28241034 PMC5328274

[7] Abrego-Guandique DM Ilori OA Caroleo MC Cannataro R Cione E Tucci P. Differential digestive stability of food-derived microRNAs: the case of miR-30c-5p and miR-92a-3p in polyfloral honey. Curr Issues Mol Biol. (2024) 46:7473–85. 10.3390/cimb46070443 39057084 PMC11276035

[8] Cione E Michelini S Abrego-Guandique DM Vaia N Michelini S Puleo V. Identification of specific microRNAs in adipose tissue affected by lipedema. Curr Issues Mol Biol. (2024) 46:11957–74. 10.3390/cimb46110710 39590304 PMC11592672

[9] Cannataro R Caroleo MC Fazio A La Torre C Plastina P Gallelli L. Ketogenic diet and microRNAs linked to antioxidant biochemical homeostasis. Antioxidants. (2019) 8:269. 10.3390/antiox8080269 31382449 PMC6719224

[10] Mar-Aguilar F Arreola-Triana A Mata-Cardona D Gonzalez-Villasana V Rodríguez-Padilla C Reséndez-Pérez D. Evidence of transfer of miRNAs from the diet to the blood still inconclusive. PeerJ. (2020) 8:e9567. 10.7717/peerj.9567 32995073 PMC7502231

